# Influence of Polysaccharides From *Polygonatum kingianum* on Short-Chain Fatty Acid Production and Quorum Sensing in *Lactobacillus faecis*

**DOI:** 10.3389/fmicb.2021.758870

**Published:** 2021-11-17

**Authors:** Min Yang, Fanying Meng, Wen Gu, Lihui Fu, Fan Zhang, Fengjiao Li, Yating Tao, Zhengyang Zhang, Xi Wang, Xingxin Yang, Jingping Li, Jie Yu

**Affiliations:** ^1^Yunnan Key Laboratory of Southern Medicine Utilization, College of Pharmaceutical Science, Yunnan University of Chinese Medicine, Kunming, China; ^2^Kunming Third People’s Hospital, Kunming, China

**Keywords:** *Lactobacillus faecis*, quorum sensing, short-chain fatty acids, polysaccharides, *Polygonatum kingianum*

## Abstract

Polysaccharide is one of the main active ingredients of *Polygonatum kingianum*, which has been proven to regulate the balance of gut microbiota. For the first time, this study focused on the regulation of polysaccharides from *Polygonatum kingianum* (PS) on *Lactobacillus faecis*, a specific probiotic in the intestinal tract. PS effectively promoted the biomass, biofilm and acetic acid production in *L. faecis* 2-84, and enhanced quorum sensing (QS) signaling. The characteristics of gene sequence were analyzed using genomics approaches, and *L. faecis* 2-84 was found to encode 18 genes that are closely related to QS and 10 genes related to short-chain fatty acids (SCFAs). Additionally, transcriptome and proteome analysis demonstrated that PS could promote the QS system of *L. faecis* by enhancing the transcription of *oppA* gene and expression of oppD protein. PS also regulated the production and metabolism of SCFAs of *L. faecis* by upregulating the expression of *ldh* and *metE* gene and adh2 protein, and downregulating the expression of *mvK* gene. In conclusion, it was speculated that PS could affect intestinal SCFAs production by affecting the QS system and SCFAs production in *L. faecis*. The present study implied that PS might have a role in promoting the growth of intestinal probiotics, where the QS system and SCFAs might be two of the important mechanisms for the probiotic activity of PS.

## Introduction

Gut microbiota remains a major research focus due to their known positive or negative effects on human health (Priyadarshini et al., 2018; Yu et al., 2018). The composition of human gut microbiota is mainly determined by genetic factors and lifestyle but altered by diet and drug usage. Polysaccharides are high molecular polymers, widely existed in edible plants. Polysaccharides that cannot be directly absorbed by the human body are converted into oligosaccharides and monosaccharides through the fermentation of gut microbiota to produce short-chain fatty acids (SCFAs) (Liu et al., 2020). SCFAs are organic fatty acids with a carbon chain of 1–6, in which the substrate and strain influence the quantity of SCFAs. Previous studies have shown that SCFAs play a key role in the regulation of host metabolism and intestinal inflammation (Hernández et al., 2019; Xiao et al., 2020). Polysaccharides also significantly impact gut microbiota through the quorum sensing (QS) system (Vishwakarma and Sirisha, 2020). Bacteria can release and exchange autoinducers (AIs), which monitor their population density and coordinate group behavior, including bacterial growth, proliferation, pathogenicity, and biofilm formation. This process, known as QS, can improve the environmental adaptability of bacteria. The signaling molecules of the QS system include autoinducing peptides (AIP), N-acylhomoserine lactones (AHL), and 4,5-dihydroxy-2,3-pentanedione (AI-2) (Whiteley et al., 2017). QS is widespread in gut microbiota and regulates its composition, thereby ultimately influencing human health. Together, gut microbiota plays a regulatory role in energy metabolism and immunity through the SCFAs metabolites and QS system. Therefore, it was speculated that SCFAs and QS might be important mediators, regulating gut microbiota and body health.

Polygonati Rhizoma has been frequently used as traditional herbal medicine or functional food and has a wide range of edible value in China. *Polygonatum kingianum* Coll. et Hemsl is an approved source of Polygonati Rhizoma recorded in the Chinese Pharmacopoeia, 2020 edition. Polysaccharides from *Polygonatum kingianum* (PS) are its major active ingredient. Our previous studies have shown that PS influences SCFAs content by regulating gut microbiota and reducing lipid metabolism disorders in rats (Yan et al., 2017; Yang et al., 2018, 2019). However, the specific mechanism through which PS regulates the SCFAs producing bacteria remains unclear. In particular, the influence of QS system, which impacts the function of gut microbiota, is largely undefined.

In order to better understand the regulation and mechanism of PS on gut microbiota, this study screened a strain of *Lactobacillus faecis*. The regulatory effects of PS on gut microbiota were observed through a single, specific and probiotic strain. *L. faecis*, which was isolated for the first time in 2013 (Endo et al., 2013). *L. faecis* has the biological characteristics of acid resistance, bile salt tolerance, adhesion to the intestines of mice, and alleviation of anxiety symptoms in mice (Su et al., 2016). However, there is no study on the QS and SCFAs production of *L. faecis*, especially by natural products from diet or functional foods. In this study, genomic, transcriptomic, and proteomic analyses of *L. faecis* were performed for the first time, and its QS system and SCFAs related mechanisms were explored, which provided references for *L. faecis* related studies. The molecular mechanism of PS, regulating the bacterial SCFAs production and QS system, were further studied, taking *L. faecis* as representative, which preliminary revealed the mechanism for the regulation of gut microbiota by PS.

## Materials and Methods

### Preparation and Characterization of Total Polysaccharides

Fresh *Polygonatum kingianum* Coll. et Hemsl rhizomes were sectioned into small pieces (2–3 cm) and placed in a juice extractor with 10 volumes of ultrapure water. The juice was sonicated for 30 min, centrifuged at 12,000 rpm and 4°C for 10 min, and the supernatants were collected. The residue was then re-extracted in 10 volumes of ultrapure water again. All the supernatants were then combined, concentrated, and precipitated in 95% ethanol at 4°C for 24 h. Precipitates were collected and dissolved in a 60°C water bath to remove ethanol, lyophilized to obtain dried polysaccharides, and stored in a desiccator for future use.

As previously described the molecular weights of PS were measured through a high-performance gel permeation chromatogram (HPGPC) (LiHui, 2019). The chemical profiles of PS were characterized through the detection of total carbohydrates, proteins, and uronic acid. Total carbohydrates were determined using the phenol-sulfuric acid method, using glucose as a standard. Protein contents were determined through Coomassie brilliant blue staining using bovine serum albumin (BSA) as a standard. The content of uronic acid was determined according to the Blumenkrantz and Asboe-Hansen method using D-glucuronic acid as a standard. The compositions of monosaccharides in PS were assessed by pre-column derivative-high performance liquid chromatography (HPLC). The quality of PS was examined from multiple perspectives.

### Bacterial Strains and Reagents

Male Sprague Dawley (SD) rats (5–6 weeks old) (Changsheng Laboratory Animal Technology Co., Ltd., Liaoning, China), weighing 180 ± 20 g, were anesthetized with sodium pentobarbital (40 mg/kg), which was administered intraperitoneally, and euthanized by cervical dislocation. The animal experiments were approved by the Animal Experiment Ethics Committee of Yunnan University of Traditional Chinese Medicine (Approval Number: R-0620180001). Rats were handled according to the guidelines set by the National Institutes of Health (NIH). Then, the rats were quickly dissected to remove their intestinal contents.

A total of 1 g of rat intestinal contents was added to aseptic saline to make a microbial suspension. The microbial suspension was diluted by a series of dilutions (such as 1:10, 1:100, 1:1,000, and 1:10,000), and 20 μL of microbial suspension was spread on the surface of MRS medium (HuanKai Technology Company, Guangdong, China). Multiple single colonies were selected after growing on MRS medium. The multiple single colonies were inoculated on selective medium (LBS medium and LC medium), and bacteria were isolated and purified using streak plate method. The yield of QS signal molecule AI-2 and metabolite SCFAs production of each single strain were determined, and the bacteria, secreting AI-2 and having stronger ability to produce SCFAs, were selected as the target strain. We finally screened the target strain *L. faecis 2-84.* The reporter strain *Vibrio harveyi* BB170 (strain BNCC337376) was purchased from Beijing Beina Biological Co., Ltd. and cultured in 2216E medium (HaiBo Technology Company, Qingdao, China). All the strains were stored at −80°C until further use.

### Growth-Curves

To determine the effects of PS on the growth of target strain, *L. faecis* 2-84 was inoculated into MRS medium, and incubated under anaerobic conditions at 37°C to reach OD_600_ = 0.6–0.8. The mixed bacterial culture (1%) was then added to MRS medium, containing different concentrations of PS (10, 20, and 30 μg/mL) under anaerobic conditions at 37°C, 180 rpm for 24 h. Optical density was measured at 600 nm using a UV-1800 Spectrophotometer (Shanghai Meipuda instrument, China) every 2 h.

### Biofilm Assay

The crystal violet method was used to quantify biofilm formation. *L. faecis* 2-84 was grown in MRS medium to OD_600_ = 0.8 and 1% of the mixed bacterial culture was treated with the varying concentrations of PS. Assays were repeated in triplicates, with sterile solutions being used as negative controls. Plates were incubated at 37°C for 4, 8, 12, 16, 20, 24, 48, 72, 96, and 120 h to allow biofilm formation. At each time point, planktonic bacteria were removed and the cells were washed with phosphate buffered saline (PBS). Adherent bacteria were fixed in methanol for 20 min, naturally air-dried, and then stained with 1% crystal violet for 30 min. Finally, 500 μL of 33% glacial acetic acid was added to each well to dissolve the crystal violet. Absorbance values were measured at 595 nm using a Variskan flash automatic microplate reader (Thermo Fisher Scientific, United States).

The characteristics of biofilm quality were preliminarily characterized using crystal violet staining (CV), and scanning electron microscopy (SEM) was used to further observe the microstructure and morphologies of biofilms after treatment with PS. Bacteria were inoculated into 12-well plates and positively charged glass slides were placed in each well to adsorb the biofilms. After 96 h incubation, wells were washed with PBS and fixed in 10% glutaraldehyde. Samples were dehydrated in a graded ethanol series, and dried in a desiccator overnight before observational analysis.

### Autoinducer-2 Bioassays

AI-2 bioassays were performed to determine the effects of PS on AI-2 production in *L. faecis* 2-84 as described by Ismail et al. (2016) with minor modifications. Samples were measured at the same OD value to eliminate the influence of bacterial numbers. Briefly, the reporter strain *V. harveyi* BB170 was cultured overnight at 30°C and 180 rpm in 2216E medium, and cells were diluted to 1:5,000. *L. faecis* 2-84 was inoculated into MRS medium and incubated under anaerobic conditions at 37°C to reach OD_600_ = 0.6–0.8. The mixed bacterial cultures (1%) were added to the MRS liquid medium, containing different concentrations of PS (administration PS group) and blank (control group) under anaerobic conditions at 37°C and 180 rpm. Bacterial solutions with ODs_600_ = 0, 0.05, 0.1, 0.15, and 0.2 were centrifuged at 12,000 rpm and 4°C for 10 min and their supernatants were mixed with *V. harveyi* BB170 at a ratio of 1:9 (v/v). Mixtures were incubated at 30°C and 180 rpm for 3 h and 200 μL/well of the bacterial solution was added into 96-well black plates, with 2216E medium used as negative control. Bioluminescence was recorded using Variskan flash automatic microplate reader (Thermo Fisher Scientific, United States). Relative luminescence units (RLU) were used to quantify the AI-2 activity (RLU = sample mean/negative control mean).

### Gas Chromatography-Mass Spectrometer Analysis of Short-Chain Fatty Acids

*L. faecis* 2-84 was inoculated into MRS medium under anaerobic conditions to OD_600_ = 0.6–0.8. Next, 1% of the mixed bacterial culture was added to MRS medium, containing different concentrations of PS (10, 20, 30 μg/mL) or AI-2 (10, 20, 30 μg/mL), under anaerobic conditions at 37°C and 180 rpm. Bacterial solutions with ODs_600_ = 0, 0.05, 0.1, 0.15, and 0.2 were centrifuged and treated with 5 volumes of acetone to remove impurities. Mixtures were centrifuged, passed through 0.2 μm filters and stored at 80°C until analysis. The standard solutions of acetate acid, propionate acid, and butyrate acid were prepared in acetone with different concentration gradients (0.005, 0.01, 0.02, 0.05, 0.1 mol/L) and filtered for testing.

An Agilent GC 7890B Gas Chromatography System (Agilent Technologies) was used to quantify the SCFAs. Samples (1 μL) were injected into the gas chromatograph equipped with an DB-WAX column (30 m × 250 μm, 0.25 μm, Agilent) using He as the gas carrier at a constant flow rate of 1 mL/min. Chromatographic conditions were as follows: split ratio 50:1, initial oven temperature 120°C, held for 0.5 min, ramped to 130°C at 8°C/min, held for 1 min, and a ramp of 20°C/min up to 200°C, held for 5 min. In the MS detector, the electron impact energy was set at 70 eV. Data were collected in the range of 50–150 atomic mass units. Samples were analyzed at least in triplicates. SCFAs were identified by comparison of their mass spectra with those held in the mass spectra library and through the comparison of their retention times with the corresponding standards. SCFAs were quantified using linear regression equations (*R*^2^ > 0.99) from standard curves.

### Multi-Omics Analysis

For genomic analysis, DNA was analyzed by BGI Health Co., Ltd. (Supplementary Material 1 for specific methods). For transcriptome analysis, RNA was analyzed by PTM Biolab Co., Ltd. (Supplementary Materials 1 2 for specific methods). For proteomic analysis, the samples were analyzed by PTM Biolab Co., Ltd. (Supplementary Material 3 for specific methods).

### Statistical Analysis

All the data presented as mean ± S.E.M. All the statistical analyses were performed using SPSS 16.0 software. A one-way ANOVA was performed for group comparisons. When assuming that the variances were equal, the LSD:1 method was used for comparisons. For non-uniform variances, the Tamhanes T2 test was performed (*^∗^P* < 0.05, *^∗∗^P* < 0.01, and *^∗∗∗^P* < 0.001).

## Results

### Preliminary Characterization of *Polygonatum kingianum*

The identification and characterization of PS had been conducted and published by our research group (LiHui, 2019; Gu et al., 2020). The average molecular weight of PS was 1.347 × 10^5^ Da determined using HPGPC. The total sugar, protein and uronic acid contents of PS were determined to be 71.12, 11.48, and 15.01%, respectively. The acid hydrolyzate derived from PS was analyzed using HPLC and consisted of mannose (35.05%), galactose (33.44%), glucose (15.06%), arabinose (6.14%), galacturonic acid (5.82%), ribose (2.38%), and rhamnose (2.1%).

### Effect of *Polygonatum kingianum* on *Lactobacillus faecis*

A total of 22 strains, having different colors, shapes and sizes, were isolated and purified from the rat intestines. In order to explore the existence of AI-2 QS system in these strains, their AI-2 activities were determined. Four strains with AI-2 activities were screened out, namely 1-43, 2-84, 2-87, and 2-122. The RLU values of these strains were 1.66, 2.37, 1.97, and 2.21, respectively. The strains with AI-2 activity were further screened to determine their SCFAs production. The acetic acid contents of these strains were 37.48, 42.62, 39.41, and 40.24, respectively. The results showed that the concentration of acetic acid, produced by the strain 2-84, was more abundant than the others. Therefore, the strain 2-84 was selected as target strain for the further study. The *16S rRNA* gene sequence of strain 2-84 was determined after bidirectional sequencing. The spliced sequence of strain 2-84 was compared with those in GenBank. The comparison results showed that the highest similarity (99.8%) of this sequence with that of *Lactobacillus faecis* AFL13-2 (NR. 1143911). Therefore, the strain 2-84 was identified as *Lactobacillus faecis*.

**FIGURE 1:**
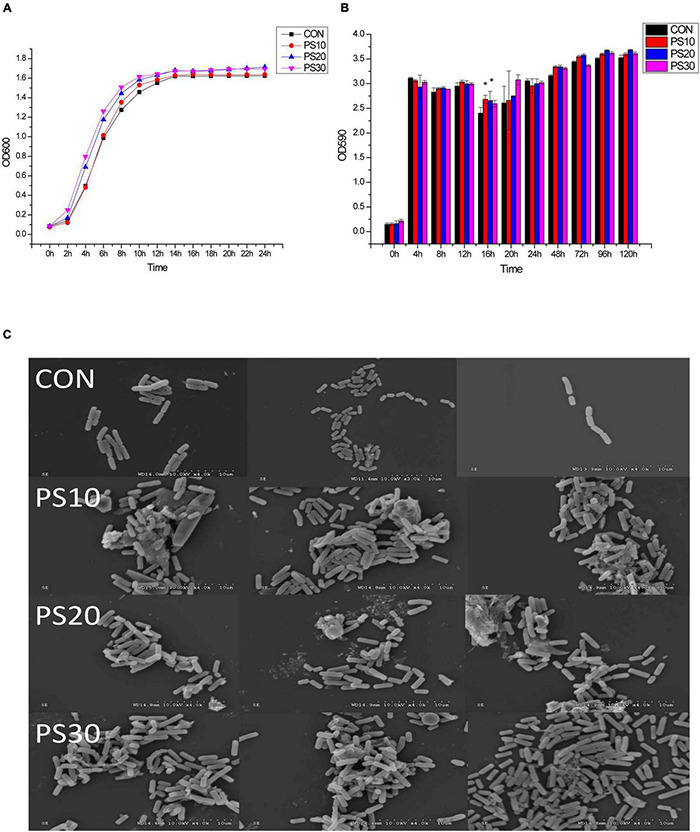
Effects of PS on the growth of *Lactobacillus faecis* 2-84. **(A)** Growth curve; **(B)** biofilm formation: **(C)** biofilm morphology; CON, control group; PS10, PS20 and PS30 represent the groups administered with 10, 20 or 30 μg/mL PS. *P < 0.05 as compared to the control group.

It was found that PS effectively promoted the growth of *L. faecis* 2-84. *L. faecis* 2-84 entered the logarithmic growth phase after about 4 h of incubation and reached the plateau phase at about 16 h. The duration to enter the logarithmic growth phase was shortened after the application of 20–30 μg/mL PS. Bacterial abundance increased by 19.03%/27.63% after 6 h and by 5.54%/4.31% after 16 h of treatment with 20/30 μg/mL PS (Figure 1A).

Qualitative and quantitative experiments on the bacterial biofilms were performed to investigate the ability of PS to promote their formation. There was a significant difference in the promotion of biofilm by 10–20 μg/mL PS at 16 h of bacterial growth, and overall PS tended to promote the biofilm production (Figure 1B). At 96 h, the levels of bacterial biofilms were stable. Then, this time point was selected to observe the biofilm structure. The SEM images of *L. faecis* 2-84 showed that the biofilms were visible and uniform (Figure 1C). In the CON group, a flat and thin biofilm was observed, but the lower levels of bacterial aggregation occurred. In the PS group, bacteria were observed on the cell climbing sheet, showing the higher levels of aggregation and proliferation.

**FIGURE 2:**
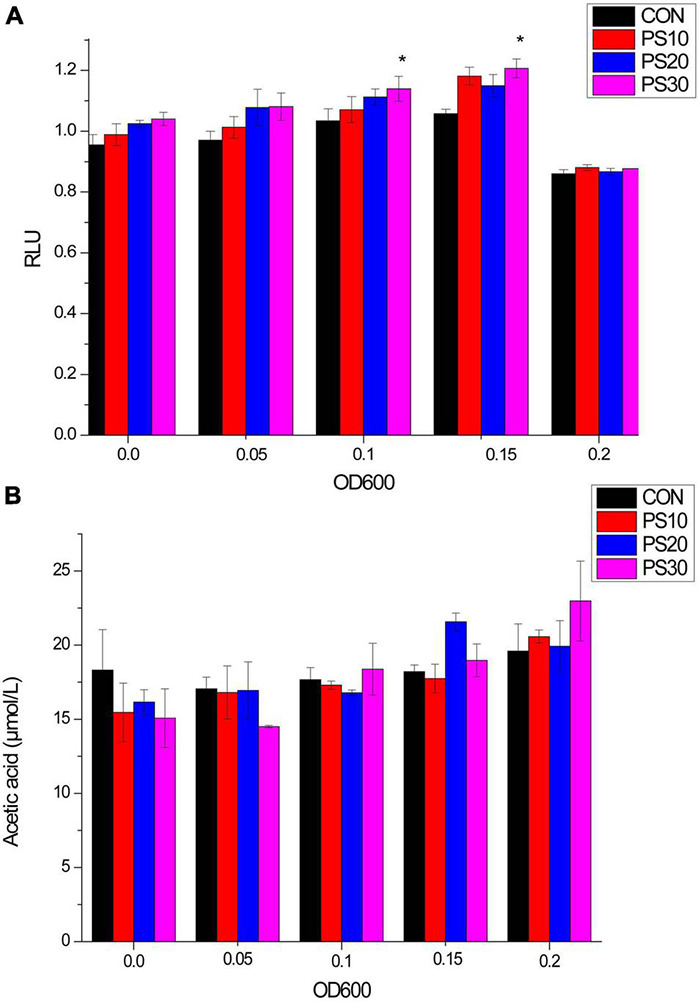
Effects of PS on AI-2 activity and acetic acid production of *Lactobacillus faecis* 2-84. **(A)** Influence of PS on AI-2 activity of *L. faecis:* **(B)** influence of PS on the acetic acid production of *L. faecis*. CON, control group; PS10, PS20, and PS30 represent PS group administered with 10, 20, and 30 μg/mL PS. *P < 0.05 as compared to the control group.

We investigated the effects of PS on AI-2. Following the PS administration, AI-2 activity increased. The secretion of signaling molecules was partially inhibited at peak environmental AI-2 concentrations (OD_600_ = 0.20) (Figure 2A).

The contents of propionic acid and butyric acid were often lower than the lower limit of the sensitivity of GC/MS determination experimental method established in this study and unstable in this part of the study. Therefore, we focused on the changes in acetic acid contents. Following the administration of PS, the acetic acid content of bacteria gradually increased. In initial experiments, the acetic acid content of the PS administration group was comparable to that of the CON group. However, with the growth of *L. faecis* 2-84, the acetic acid content of the administration group was higher than that of the CON group. When the bacterial growth reached OD_600_ = 0.20, the acetic acid content of the administration groups was higher than that of the CON group. Among them, 30 μg/mL PS displayed the best potent effect on the SCFAs production (Figure 2B).

### Genomic Analysis of *Lactobacillus faecis*

**FIGURE 3:**
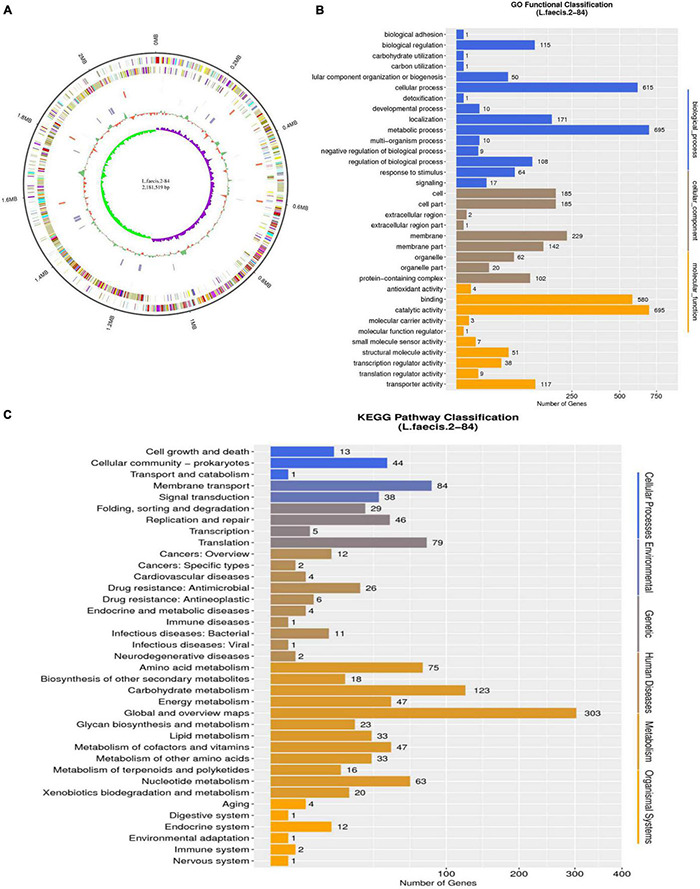
Genomic data analysis of *Lactobacillus faecis* 2-84. **(A)** Circular representation of genome: **(B)** GO annotation results; **(C)** KEGG annotation results.

The genome of *L. faecis* 2-84 by *de novo* sequencing was evaluated and the genes related to QS system and SCFA production in *L. faecis* 2-84 were explored. This provided a preliminary basis for the subsequent exploration of changes in gene transcription and translation in the QS system and SCFAs pathways and genomic sequences have been deposited in NCBI under accession number PRJNA771777. The whole genome of *L. faecis* 2-84 contained a circular chromosome with a fragment length of 1,886,946 bp and a GC content of 40.45%. This strain has 1208 genes annotated by the GO database, of which the catalytic activities and metabolic processes accounted for the largest number of annotations. In the KEGG database, 74.14% of the genes were assigned to metabolism pathways, whereas 19 and 3 pathways were related to SCFAs and QS system (Figure 3).

#### Quorum Sensing Gene Mining

Three pathways related to QS system were screened in the KEGG database [Quorum sensing (map02024); Biofilm formation—*Vibrio cholerae* (map05111); Biofilm formation—*Escherichia coli* (map02026)], involving a total of 47 genes. Based on the results of KEGG pathway annotation, the QS activity of *L. faecis* 2-84 was mined (Table 1). From the genetic level analysis, the genes closely related to QS in *L. faecis* 2-84 included: *luxs*, a gene related to AI-2 QS (Zhang et al., 2019); *oppA* (*–B, –C, –D*), a gene related to oligopeptide systems (Zheng et al., 2018); *ABC.PE.A*, a gene related to ABC transporters that provide energy for transmembrane transport; the QS signal molecular transport-related genes *SecA, –E, –G, –Y*; nisin (QS signal molecules) related genes *nisE* (*–F, –K, –R*); and biofilm formation related genes to ptsG, glgA, glgC, glgP, etc. (Merino et al., 2012).

**TABLE 1:** Quorum sensing related genes of *L. faecis* 2-84.

**Gene name**	**ID**	**Gene function description**
*luxS*	*L. faecis*.2-84GL000811	s-ribosylhomocysteine lyase
*oppA*, *mppA*	*L. faecis*.2-84GL000184; *L. faecis*.2-84GL000196; *L. faecis*.2-84GL000854; *L. faecis*.2-84GL001142; *L. faecis*.2-84GL001682	Oligopeptide transport system substrate-binding protein
*oppB*	*L. faecis*.2-84GL000197	Oligopeptide transport system permease protein
*oppC*	*L. faecis*.2-84GL000198	Oligopeptide transport system permease protein
*oppD*	*L. faecis*.2-84GL000199	Oligopeptide transport system ATP- binding protein
*oppF*	*L. faecis*.2-84GL000200	Oligopeptide transport system ATP- binding protein
*secA*	*L. faecis*.2-84GL000797	Preprotein translocase subunit SecA
*secE*	*L. faecis*.2-84GL001622	Preprotein translocase subunit SecE
*secG*	*L. faecis*.2-84GL000827	Preprotein translocase subunit SecG
*secY*	*L. faecis*.2-84GL000356	Preprotein translocase subunit SecY
*yajC*	*L. faecis*.2-84GL000597	Preprotein translocase subunit YajC
*nisE*, *spaE, cprB*, *epiE*	*L. faecis*.2-84GL001829	Lantibiotic transport system permease protein
*nisF*, *spaF, cprA*, *epiF*	*L. faecis*.2-84GL001830	Lantibiotic transport system ATP- binding protein
*nisK*, *spaK*	*L. faecis*.2-84GL001831	Two-component system, OmpR family, lantibiotic biosynthesis sensor histidine kinase NisK/SpaK
*nisR*, *spaR*	*L. faecis*.2-84GL001832	Two-component system, OmpR family, lantibiotic biosynthesis response regulator NisR/SpaR
*ABC. PE.A*	*L. faecis*.2-84GL001958; *L. faecis*.2-84GL001959	Peptide/nickel transport system ATP-binding protein
*ABC. PE.S*	*L. faecis*.2-84GL001533; *L. faecis*.2-84GL001951; *L. faecis*.2-84GL001952; *L. faecis*.2-84GL001953; *L. faecis*.2-84GL001955;	Peptide/nickel transport system substrate-binding protein
*ABC. PE.P*	*L. faecis*.2-84GL001956; *L. faecis*.2-84GL001957	Peptide/nickel transport system permease protein
*yidC*, *spoIIIJ, OXA1*, *ccfA*	*L. faecis*.2-84GL001315; *L. faecis*.2-84GL001986	YidC/Oxa1 family membrane protein insertase
*blpA*, *lagD*	*L. faecis*.2-84GL000787	ATP-binding cassette, subfamily C, bacteriocin exporter
*ftsY*	*L. faecis*.2-84GL001133	Fused signal recognition particle receptor
*lacD*	*L. faecis*.2-84GL001356	Tagatose 1,6-diphosphate aldolase
*SRP54*, *ffh*	*L. faecis*.2-84GL001131	Signal recognition particle subunit SRP54
*PTS-Glc-EIIC, ptsG*	*L. faecis*.2-84GL000262	PTS system, glucose-specific IIC component
*glgC*	*L. faecis*.2-84GL000466; *L. faecis*.2-84GL000467	Glucose-1-phosphate adenylyltransferase
*glgA*	*L. faecis*.2-84GL000468	Starch synthase
*PYG*, *glgP*	*L. faecis*.2-84GL000469	Glycogen phosphorylase
*wecB*	*L. faecis*.2-84GL000405	UDP-N-acetylglucosamine 2-epimerase (non-hydrolyzing)
*rpoN*	*L. faecis*.2-84GL000820	RNA polymerase sigma-54 factor
*tagA*, *tarA*	*L. faecis*.2-84GL001650	N- acetylglucosaminyldiphosphoundecaprenol N-acetyl-beta- D-mannosaminyltransferase
*yidC*, *spoIIIJ, OXA1*, *ccfA*	*L. faecis*.2-84GL001315	YidC/Oxa1 family membrane protein insertase
*rgg3*	*L. faecis*.2-84GL000025	HTH-type transcriptional regulator, SHP3-responsive repressor
*sinR*	*L. faecis*.2-84GL001772	XRE family transcriptional regulator, master regulator for biofilm formation

#### Short-Chain Fatty Acids Production Gene Mining

Based on KEGG pathway annotation, the SCFA-related pathways were screened in the genomic datasets. Among them, the acetic acid, propionic acid, and butyric acid were jointly involved in two pathways [metabolic pathways (map01100); carbohydrate digestion and absorption (map04973)], acetic acid and propionic acid were jointly involved in three pathways [propanoate metabolism (map00640); microbial metabolism in diverse environments (map01120); degradation of aromatic compounds (map01220)], acetic acid alone was involved in 12 pathways (map00010; map00430; map00440; map00620; map00630; map006604; map00680; map00720; map00908; map00920; map01110; and map01200), and propionic acid and butyric acid were involved only in a single pathway [nicotinate and nicotinamide metabolism (map00760); butanoate metabolism (map00650), respectively]. The genes closely related to SCFAs were further screened, which included: *ackA* and *pta* genes related to the pathway of acetyl-CoA to synthesize acetate (Schütze et al., 2020); *pdhA, –B*, and *acyP* genes related to the pathway of pyruvate to produce acetate (Landi et al., 1986; Tymecka-Mulik et al., 2017); *ldh* gene related to propionic acid metabolism (Zhao et al., 2019); and *adhE* gene related to butyrate production (Loder et al., 2015; Table 2).

**TABLE 2:** Short chain fatty acid related genes of *L. faecis* 2-84.

**Gene name**	**ID**	**Gene function description**
*ackA*	*L. faecis*.2-84GL001598; *L. faecis*.2-84GL001721; *L. faecis*.2-84GL001810	Acetate kinase
*E2.3.1.8*, *pta*	*L. faecis*.2-84GL000450	Phosphate acetyltransferase
*PDHA, pdhA*	*L. faecis*.2-84GL001422	Pyruvate dehydrogenase E1 component alpha subunit
*PDHB*, *pdhB*	*L. faecis*.2-84GL001421	Pyruvate dehydrogenase E1 component beta subunit
*DLAT*, *aceF, pdhC*	*L. faecis*.2-84GL001420	Pyruvate dehydrogenase E2 component (dihydrolipoamide acetyltransferase)
*DLD*, *lpd*, *pdhD*	*L. faecis*.2-84GL001419	Dihydrolipoamide dehydrogenase
*acyP*	*L. faecis*.2-84GL001314	Acylphosphatase
*cysK*	*L.* ..*faecis.*2-84GL000047; *L. faecis.*2-84GL001497	Cysteine synthase
*LDH*, *ldh*	*L. faecis*.2-84GL000598	L-lactate dehydrogenase
*adhE*	*L. faecis*.2-84GL000254	Acetaldehyde dehydrogenase/alcohol dehydrogenase

### Effects of *Polygonatum kingianum* on the *Lactobacillus faecis* Transcriptome

**FIGURE 4:**
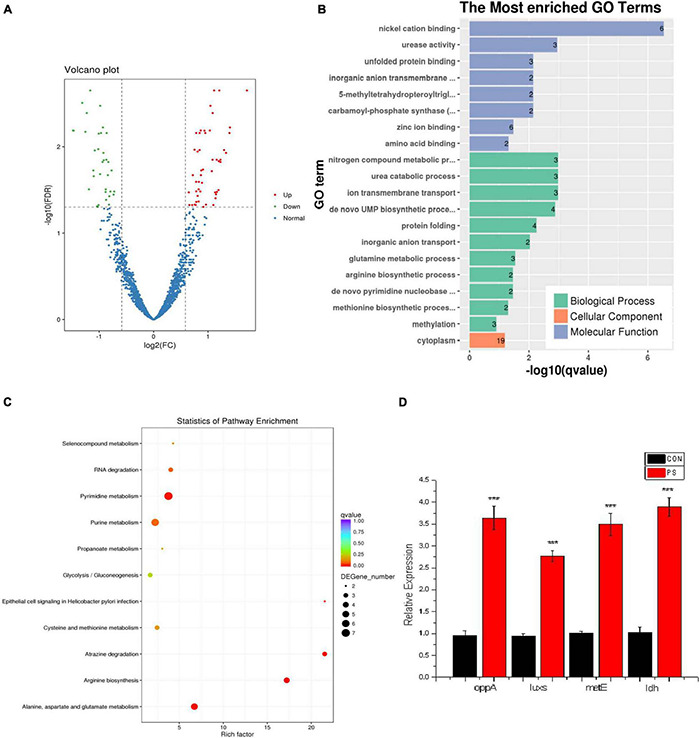
Transcriptome data analysis of *Lactobacillus faecis* 2-84. **(A)** Differential expression volcano plot; **(B)** GO enrichment results; **(C)** differential gene KEGG enrichment statistical results; **(D)** expression levels of key genes in the *Lactobacillus faecis* transcriptome. ***P < 0.001 as compared to the control group.

RNA sequences have been deposited in NCBI under accession number PRJNA771790. According to orthogonal experiments, the CON and PS administration groups (20 μg/mL) were selected for transcriptome and proteomic analysis when the OD_600_ of bacterial solution reached 0.05 (Supplementary Material 2). From transcriptome data, the quantitative analysis of transcripts in the CON group and PS group led to a total of 85 differential transcripts being screened, including 50 up-regulated genes and 35 down-regulated genes. The distribution of differential genes in GO was further investigated using enrichment analysis. The enriched differential transcripts were focused on the nickel cation binding, urease activity, and nitrogen compound metabolic process in GO database. The enriched differential transcripts were annotated to 11 KEGG pathways, focusing on the metabolism of alanine, aspartate, and glutamate and biosynthesis of arginine (Figures 4A–C).

A total of 43 differential transcripts were annotated to KEGG pathways, involving one QS system (map02024) and 11 SCFA pathways (map01100; map00640; map01120; map01220; map00010; map00440; map00620; map00630; map00680; map01110; map00650). Based on KEGG pathway annotation results, the differential transcripts related to QS and SCFAs were screened. Among them, the QS-related gene *oppA* was up-regulated, 11 genes related to SCFAs were up-regulated, and 10 genes were down-regulated (Table 3).

**TABLE 3:** Quorum sensing and short chain fatty acid related transcription of *L. faecis* 2-84 in transcriptomic data.

**Type**	**Gene name**	**ID**	**Gene function description**	
QS	*oppA*, *mppA*	*L. faecis*.2-84GL000854	Oligopeptide transport system substrate-binding protein	up-regulation
SCFAs	*ureA*	*L. faecis*.2-84GL000171	Urease subunit gamma	Up-regulation
	*ureB*	*L. faecis*.2-84GL000172	Urease subunit beta	Up-regulation
	*ureC*	*L. faecis*.2-84GL000173	Urease subunit alpha	Up-regulation
	*metE*	*L. faecis*.2-84GL000334; *L. faecis*.2-84GL001689	5-methyltetrahydropteroyltriglutamate–homocysteine methyltransferase	Up-regulation
	*guaA*, *GMPS*	*L. faecis*.2-84GL000380	GMP synthase (glutamine-hydrolyzing)	Up-regulation
	*LDH*, *ldh*	*L. faecis*.2-84GL000598	L-lactate dehydrogenase	Up-regulation
	*glnA*, *GLUL*	*L. faecis*.2-84GL001155	Glutamine synthetase	Up-regulation
	*dapE*	*L. faecis*.2-84GL001228	Succinyl-diaminopimelate desuccinylase	Up-regulation
	*puuE*	*L. faecis*.2-84GL001513	4-aminobutyrate aminotransferase	Up-regulation
	*ndk*, *NME*	*L. faecis*.2-84GL001684	Nucleoside-diphosphate kinase	Up-regulation
	*E1.1.1.1*, *adh*	*L. faecis*.2-84GL001691	Alcohol dehydrogenase	Up-regulation
	*E3.1.3.41*	*L. faecis*.2-84GL000567	4-nitrophenyl phosphatase	Down-regulation
	*E2.7.1.36*, *MVK, mvaK1*	*L. faecis*.2-84GL000927	Mevalonate kinase	Down-regulation
	*pyrB*, *PYR2*	*L. faecis*.2-84GL001026	Aspartate carbamoyltransferase catalytic subunit	Down-regulation
	*URA4*, *pyrC*	*L. faecis*.2-84GL001027	Dihydroorotase	Down-regulation
	*carA*, *CPA1*	*L. faecis*.2-84GL001028	Carbamoyl-phosphate synthase small subunit	Down-regulation
	*carB*, *CPA2*	*L. faecis*.2-84GL001029	Carbamoyl-phosphate synthase large subunit	Down-regulation
	*cmk*	*L. faecis*.2-84GL001106	CMP/dCMP kinase	Down-regulation
	*ycsE*, *yitU*, *ywtE*	*L. faecis*.2-84GL001213	5-amino-6-(5-phospho-D-ribitylamino)uracil phosphatase	Down-regulation
	*xpt*	*L. faecis*.2-84GL001375	Xanthine phosphoribosyltransferase	Down-regulation
	*pyrG*, *CTPS*	*L. faecis*.2-84GL001401	CTP synthase	Down-regulation

Based on the transcriptome data, the *oppA* gene was selected in the oligopeptide system for verification. On the other hand, using *in vitro* experiments, PS could effectively promote the activity of AI-2, the *luxs* gene was selected in the AI-2 QS system for verification. From genes involved in the metabolism of SCFAs, *metE* and *ldh* genes, which were related to acetic acid and propionic acid metabolism, respectively, were selected for verification. These selected transcripts were verified by real-time PCR analysis. PS could promote the expression of *oppA* in the oligopeptide system and the *luxs* gene in the AI-2 system. PS also enhanced the expression of acetic acid-related gene *metE* and propionic acid metabolism-related gene *ldh* (Figure 4D). It was confirmed that PS promoted QS system and SCFAs production by enhancing the expression of these key nodes.

### Effects of *Polygonatum kingianum* on the *Lactobacillus faecis* Proteome

**FIGURE 5:**
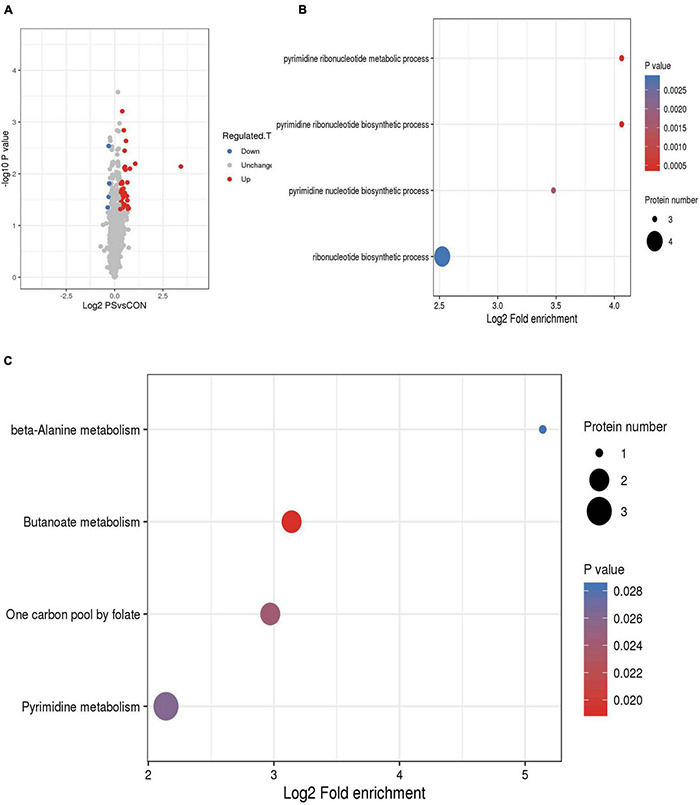
Proteome data analysis of *Lactobacillus faecis* 2-84. **(A)** Circular representation of genome; **(B)** GO annotation; **(C)** KEGG annotation.

Proteomic analysis was used to evaluate the effects of PS on *Lactobacillus faecis* genes expression. The mass spectrometry proteomics data have been deposited to the ProteomeXchange Consortium via the PRIDE partner repository with the dataset identifier PXD029210. By screening the differential expression of proteins within each group, 43 proteins were identified, including 39 up-regulated proteins and 4 down-regulated proteins. Differential proteins were enriched in GO database (pyrimidine ribonucleotide metabolic process; pyrimidine ribonucleotide biosynthetic process; pyrimidine nucleotide biosynthetic process; and ribonucleotide biosynthetic process). Ribonucleotide is a basic unit that constitutes the genetic structure and information of an organism. It was found that the differential proteins are associated with the production of ribonucleotide. The differential proteins were mainly enriched in four pathways (Butanoate metabolism, One carbon pool by folate, Pyrimidine metabolism, and beta-Alanine metabolism). Pyrimidine metabolism is the pathway with the highest number of differential proteins (Figure 5).

Based on these results, one single QS system KEGG pathway (map02024) and 3 SCFAs KEGG pathways (map01100; map01120; and map00650) were shown to be involved. Based on the results of KEGG pathway annotation, the differential proteins related to QS and SCFAs were screened (Table 4). Two proteins related to QS were up-regulated, including oppD related to the oligopeptide system. A total of 5 differential proteins related to SCFAs were up-regulated, including adh2 related to the production of acetic acid and butyric acid and pyrimidine biosynthetase pyr C, pyr R, pyr DB involved in basal bacterial metabolism.

**TABLE 4:** Quorum sensing and short chain fatty acid related transcription of *L. faecis* 2-84 in the proteomic.

**Type**	**Gene name**	**ID**	**Gene function description**	
QS	*oppD*	*L. faecis*.2-84GL001959	Oligopeptide transport ATP-binding protein OppD OS = *Lactococcus lactis* subsp. cremoris (strain SK11) OX = 272622 GN = oppD PE = 3 SV = 1	Up-regulation
	—	*L. faecis*.2-84GL001719	ABC transporter ATP-binding protein [Lactobacillus murinus]	Up-regulation
SCFAs	*adh2*	*L. faecis*.2-84GL000254	Aldehyde-alcohol dehydrogenase 2 OS = Entamoeba histolytica OX = 5759 GN = ADH2 PE = 1 SV = 1	Up-regulation
	*pyrR*	*L. faecis*.2-84GL001024	Bifunctional protein PyrR OS = Lactobacillus salivarius (strain UCC118) OX = 362948 GN = pyrR PE = 3 SV = 1	Up-regulation
	*pyrC*	*L. faecis*.2-84GL001027	Dihydroorotase OS = Lactobacillus salivarius (strain UCC118) OX = 362948 GN = pyrC PE = 3 SV = 1	Up-regulation
	*pyrDB*	*L. faecis*.2-84GL001030	Dihydroorotate dehydrogenase B [NAD(+)], catalytic subunit OS = *Lactococcus lactis* subsp. cremoris (strain MG1363) OX = 416870 GN = pyrDB PE = 1 SV = 2	Up-regulation
	—	*L. faecis*.2-84GL001513	Isoleucine 2-epimerase OS = Lactobacillus buchneri OX = 1581 PE = 1 SV = 1	Up-regulation

## Discussion

**FIGURE 6:**
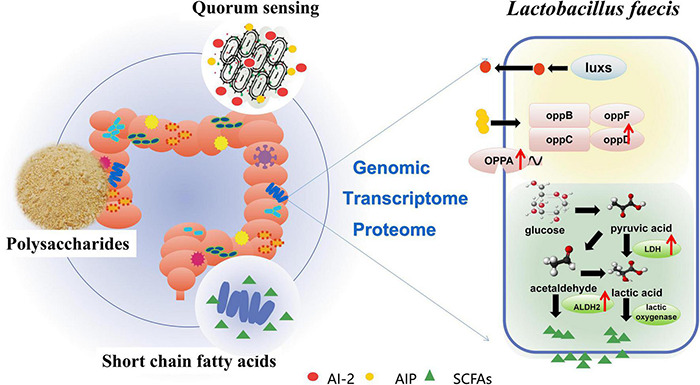
Proposed mechanism of PS on SCFAs production and QS in *Lactobacillus faecis*.

In this study, a probiotic strain *Lactobacillus faecis* 2-84 with AI-2 activity and high SCFAs production was isolated from the intestinal tract of rats. The effects of PS on *L. faecis* were further explored. It was found that PS could promote the growth of *L. faecis* 2-84, enhance the formation of bacterial biofilms, promote the activity of AI-2, and increase the concentration of acetic acid in the environment. The genome analysis of *L. faecis* 2-84 showed three KEGG pathways related to QS system and 19 KEGG pathways related to SCFAs metabolism. The whole genome information of *L. faecis* has been reported for the first time in this study. The QS system and SCFAs related genes of *L. faecis* 2-84 were explored to provide a reference for the genetic studies of *L. faecis* (Figure 6).

The QS system, as a mean of communication among bacteria in gut microbiota, is closely related to the health of host and bacterial metabolism. Using multi-omics sequencing technology, the mechanism of PS, promoting the QS system of *L. faecis* 2-84, was explored. The QS-related genes were annotated in the genome dataset and the transcriptomic and proteomic data of *L. faecis* 2-84 was analyzed. PS promoted the QS system of *L. faecis* 2-84 mainly through QS (map02024), promoting the transcription and expression of *opp*D. Opp system is a QS oligopeptide transport system, which belongs to the ATP binding box ABC transport superfamily. Oligopeptides internalized by OPP transporters play a key role in bacterial signal transduction and virulence (Zheng et al., 2018; Dubois et al., 2019). For the first time, we reported the existence of the Opp system in *L. faecis*. In addition, the presence of AI-2 (signal molecule of the LuxS/AI-2 quorum sensing system) was detected in the *in vitro* experiments. The *luxs* gene was also annotated in the genomics data; s-ribosylhomocysteine lyase (luxS) promotes the formation of signal molecule AI-2, which is an important synthetic protein in the LuxS/AI-2 QS system. However, other genes in the LuxS/AI-2 QS system could not be annotated by the transcriptomic and proteomic data due to their low expression levels. Therefore, further experiments are needed to explore the LuxS/AI-2 QS system in *L. faecis*.

As an important bioactive ingredient in traditional herbal medicine, polysaccharides cannot be directly absorbed by the human body. They are mainly converted into more easily absorbed glucose, fructose, and rhamnose, etc. by the fermentation of gut microbiota, and produce SCFA (Zhao et al., 2021). The association between PS and SCFAs was analyzed based on multi-omics sequencing technologies. Three related genes (inulosucrase, *inuJ*; Glucose-6-phosphate isomerase, *gpi*; maltose-6′-phosphate glucosidase, *glvA*) were annotated to the starch and sucrose metabolism pathway (map00500) in the *L. faecis* 2-84 genomic data, indicating that these enzymes might be involved in the hydrolysis of PS. The genes *pdhB, pdhA, pdhC, pdhD, ldh*, and *adhE* in the Glycolysis/Gluconeogenesis pathway are closely related to the SCFAs production, while Glycolysis/Gluconeogenesis pathway (map00010) is involved in the subsequent metabolism of map00500. Genomic data indicate that PS might generate SCFAs through the map00500 and map00010 pathways.

On the other hand, 11 KEGG pathways related to SCFAs production were annotated in the transcriptomic data and 3 KEGG pathways related to SCFAs production were annotated in the proteomics data. The bioinformatics analysis showed that these pathways were involved in metabolic processes, microbial metabolism in diverse environments, and butanoate metabolism in both the transcriptomic and proteomic datasets, indicating their involvement in the ability of PS to regulate the SCFAs production. In the process of acetic acid production and metabolism, PS up-regulated the expression of *ldh* and *metE* genes, and adh2 protein, and down-regulated the expression of the *mvk* gene. L-lactate dehydrogenase (LDH) is an important enzyme during lactic acid production in bacteria. Lactic acid concentrations are closely related to bacterial pyruvate metabolism, which regulates bacterial acetic acid production and propionic acid metabolism (Gaspar et al., 2014)0.5-methyltetrahydropteroyltriglutamate–homocysteine methyltransferase (metE) can increase the tolerance of *Escherichia coli* to acetic acid and increase the concentration of acetic acid in the environment (Mordukhova and Pan, 2013). Mevalonate kinase (mvk) is an ATP-dependent rate-limiting enzyme in mevalonate biosynthesis, the down-regulation of which promotes the synthesis of mevalonate (McClory et al., 2019). In the metabolic pathway of butyric acid production, PS promoted the expression of aldehyde-alcohol dehydrogenase 2 (adh2), which was annotated to butanoate metabolism pathway (map00650). In addition, adh2 is also related to the production of acetic acid, where the knockout of the *adh*2 gene could significantly decrease the acid content of yeast (YanZun, 2009).

This study is the first to analyze the effects of PS on the transcription and translation of *L. faecis*, revealing novel mechanisms for the regulation of *L. faecis* by PS. Previous studies usually focused on the inhibitory effects of natural products on the QS of pathogenic bacteria. For the first time, this study investigated the effects of natural products on the probiotic QS system, opening up new avenues of research between natural products and QS. The mechanisms of action of polysaccharides remain a research hot-spot. We revealed a novel relationship between the polysaccharides and gut microbiota (through QS system and SCFAs production). Polysaccharides are complex in chemical components, and their activities on gut microbiota are even more complex. We analyzed the mechanisms of PS *in vitro*, using *L. faecis* as an example, for the first time. However, the relative abundance of *L. faecis* in gut microbiota was relatively small. The mechanisms through which polysaccharides affect gut microbiota through QS and SCFAs still require further investigations.

## Data Availability Statement

The datasets presented in this study can be found in online repositories. The genomic data presented in the study are deposited in the NCBI repository, accession number PRJNA771777. The transcriptome data presented in the study are deposited in the NCBI repository, accession number PRJNA771790. The Proteome data presented in the study are deposited in the ProteomeXchange Consortium via the PRIDE partner repository, accession number PXD029210.

## Author Contributions

MY: conceptualization, methodology, writing—review, and editing. FM: methodology and validation. WG: resources and methodology. LF: investigation and data curation. FZ: methodology and formal analysis. FL: preparation and data curation. YT: data curation and visualization. ZZ: software and validation. XY: reviewing and editing. JL: conceptualization and supervision. JY: resources, conceptualization, and project administration. All authors contributed to the article and approved the submitted version.

## Conflict of Interest

The authors declare that the research was conducted in the absence of any commercial or financial relationships that could be construed as a potential conflict of interest.

## Publisher’s Note

All claims expressed in this article are solely those of the authors and do not necessarily represent those of their affiliated organizations, or those of the publisher, the editors and the reviewers. Any product that may be evaluated in this article, or claim that may be made by its manufacturer, is not guaranteed or endorsed by the publisher.
