# Supplementary material for: Influence of Polysaccharides From Polygonatum kingianum on Short-Chain Fatty Acid Production and Quorum Sensing in Lactobacillus faecis
Source: Front Microbiol. 2021 Nov 17;12:758870. doi: 10.3389/fmicb.2021.758870 (PMC8635744; doi:10.3389/fmicb.2021.758870)
Supplement: Supplementary file 1 [file Data_Sheet_1.zip › Supplementary Material 2.doc]

Supplementary Material 2

**(Transcriptome analysis method)**

1 Determination of optimal drug delivery conditions

In order to select the optimal drug administration conditions, according to the previous investigation results, we take the AI-2 activity of the strain as the index, the factors of drug concentration (Factor A), OD_600_ (Factor B) and incubation time with *V. harveyi* BB170(Factor C) were selected to perform three-factor and four-level orthogonal experiment.

**Table S2** Factor and level table

| level | Factor A（μg/mL） | FactorB（OD_600_） | Factor C（h） |
| --- | --- | --- | --- |
| 1 | NA | 0.05 | 0 |
| 2 | 10 | 0.10 | 1 |
| 3 | 20 | 0.15 | 2 |
| 4 | 30 | 0.20 | 3 |

According to the conditions of Table S4, the orthogonal experiment was carried out to determine the AI-2 activity of the strain RLU (RLU = the average value of the sample / the average value of the negative control). The average value of each sample was determined for 3 times, and the experimental results were as shown in Table S5. The optimal administration condition was A3B1C2. According to the results of orthogonal experiment, the CON group and the PS group (20 μg/mL) was set, and send samples for transcriptome and proteome analysis when the bacterial solution reached OD_600_=0.05.

**Table S3** Orthogonal experimental analysis table

| **number** | **group** | **A** | **B** | **C** | RLU value |
| --- | --- | --- | --- | --- | --- |
| 1 | A1B1C1 | 1 | 1 | 1 | 1.088 |
| 2 | A1B2C2 | 1 | 2 | 2 | 1.24 |
| 3 | A1B3C3 | 1 | 3 | 3 | 1.12 |
| 4 | A1B4C4 | 1 | 4 | 4 | 0.76 |
| 5 | A2B1C2 | 2 | 1 | 2 | 1.23 |
| 6 | A2B2C1 | 2 | 2 | 1 | 1.13 |
| 7 | A2B3C4 | 2 | 3 | 4 | 0.74 |
| 8 | A2B4C3 | 2 | 4 | 3 | 0.836 |
| 9 | A3B1C3 | 3 | 1 | 3 | 1.16 |
| 10 | A3B2C4 | 3 | 2 | 4 | 0.95 |
| 11 | A3B3C1 | 3 | 3 | 1 | 1.14 |
| 12 | A3B4C2 | 3 | 4 | 2 | 1.12 |
| 13 | A4B1C4 | 4 | 1 | 4 | 1.10 |
| 14 | A4B2C3 | 4 | 2 | 3 | 1.11 |
| 15 | A4B3C2 | 4 | 3 | 2 | 1.10 |
| 16 | A4B4C1 | 4 | 4 | 1 | 0.92 |
|  | k1 | 4.21 | 4.57 | 4.28 |  |
|  | k2 | 3.94 | 4.43 | **4.69** |  |
|  | k3 | 4.36 | 4.10 | 4.23 |  |
|  | k4 | 4.23 | 3.63 | 3.54 |  |

2 Bacterial RNA extraction

Set the bacteria solution without PS as the control group (CON group), and the bacteria solution with PS added as the administration group (PS group), the target strain treated with and without PS (20 μg/mL) was used for transcriptome and proteomic analysis. Briefly, target strain was inoculated into MRS medium. Then 1% mixed bacterial culture was added to MRS medium containing different concentrations of PS (0 μg/mL, 20 μg/mL), incubated under anaerobic conditions at 37°C, 180 rpm. Take the bacterial solution with OD_600_= 0.05 as the samples. Three parallel samples for each group and were processed in the same manner. Collect the bacterial cells and wash them repeatedly with sterile 1 × PBS. Finally, transfer the bacterial cells to a -80°C refrigerator for storage.

Bacterial RNA was isolated using a bacterial total RNA extraction kit (Cat. No. DP441, Tiangen, China). RNA concentration was measured using Qubit® RNA Assay Kit in Qubit® 2.0 Flurometer (Life Technologies, CA, USA). RNA purity was checked using the NanoPhotometer® spectrophotometer (IMPLEN, CA, USA) . RNA integrity was assessed using the RNA Nano 6000 Assay Kit of the Bioanalyzer 2100 system (Agilent Technologies, CA, USA). An A_260_/A_280_ ratio of at least 2.0 was considered appropriate for the experiments.

3 Illumina library construction and sequencing

The RNA samples were processed for Transcriptome sequencing analysis by PTM Biolab Co., Ltd. (Hangzhou, China). A total amount of 3 g RNA per sample was used as input material for the RNA sample preparations. Sequencing libraries were generated using NEBNext Ultra RNA Library Prep Kit for Illumina (NEB, USA) following manufacturer s recommendations and index codes were added to attribute sequences to each sample. The clustering of the index-coded samples was performed on a cBot Cluster Generation System using TruSeq PE Cluster Kit v3-cBot-HS (Illumia) according to the manufacturer’s instructions. After cluster generation, the library preparations were sequenced on an Illumina Hiseq2500/X platform and 125/150 bp paired-end reads were generated.

4 RNA Seq data analysis

The reads were aligned with the genome sequence of xxx (NCBI accession number: NC_XXX). Index of the reference genome was built using Bowtie v2.2.3 and paired-end clean reads were aligned to the reference genome using TopHat v2.0.12. Cuffquant and cuffnorm (v 2.2.1) was used to calculate FPKMs of genes in each sample. Gene FPKMs were computed by summing the FPKMs of transcripts in each gene group. For the detection of differentially expressed genes, the analysis, including data normalization, was performed with the SARTools R package based on the DESeq2 package, with default parameters.

5 Quantitative real-Time PCR for Detection of Relative Gene Expression

Based on the results of KEGG annotation, the key transcripts related to QS and SCFAs were screened out of the differential transcripts. Bacterial samples (three biological replicates in each group) were extracted from the CON group and the PS group respectively, and real-time quantitative PCR (real-time quantitative PCR, q-PCR) to verify relevant transcripts.

The cultured bacteria cells were pelleted, and the total RNA was next prepared using Eastep® Super Total RNA Extraction Kit kit(Promega, Shanghai, China). RNA integrity was determined according to OD_260/280_ ratio and around 500 ng of RNA samples were amplified to cDNA using GoScriptTM Master Mix kit (Promega, Shanghai, China). After appropriate dilution, the reverse-transcribed cDNA was further amplified to target gene fragment using SYBR green Premix Ex TaqTM kit (TaKaRa, Dalian, China). The 25 μL singleplex reactions were prepared on ice and contained 2.5 μL template DNA, 100 nM each primer (HPLC purified), and a 1 concentration of the TaqMan Universal PCR master mixture. All the primers were designed according to the genome sequences (Table S3). The PCR amplification protocol were as follows: an initial denaturation step at 95℃ for 15 min, followed by 44 cycles of 95℃ for 15 s and 56℃ for 30 s.

**Table S1** [primer](C:/Program%20Files%20(x86)/Youdao/Dict/8.9.3.0/resultui/html/index.html#/javascript:;) [sequence](C:/Program%20Files%20(x86)/Youdao/Dict/8.9.3.0/resultui/html/index.html#/javascript:;)

| Gene name | ID | [base](C:/Program%20Files%20(x86)/Youdao/Dict/8.9.3.0/resultui/html/index.html#/javascript:;) [sequence](C:/Program%20Files%20(x86)/Youdao/Dict/8.9.3.0/resultui/html/index.html#/javascript:;)(5'→3)' |
| --- | --- | --- |
| 16srRNA | 16srRNA-F | ATGATGCATAGCCGAGTTGAGAGAC |
|  | 16srRNA-R | TTCCCTACTGCTGCCTCCC |
| oppA | oppA-F | ACTTCACCAGACTAACAGCCGAAC |
|  | oppA-R | TAGCGACCCACCTCCAGCAAC |
| luxs | luxS-F | GCAGTCAAAGCACCTTAT |
|  | luxS-R | GTCACGGTAGTTTCCACA |
| ldh | LDHldh-F | ATGTTGGCGTAACTGTCG |
|  | LDHldh-R | CGTTGTAAGATCGTCGTG |
| metE | metE-F | ATCCGTTTTACTTCAGACC |
|  | metE-R | TAGCGACATTTTCCTCAC |

The main thing to note is that after the correlation analysis of the transcriptome data, it is found that the correlation between PS-2 and other samples is very poor (r=0.8755~0.9289), and the biological repeatability is very poor (PS-1 vs PS-2: r=0.9140; PS-2 vs PS-3: r=0.8755). For the rigor of the data, we excluded this sample in the subsequent difference analysis. After excluding the PS-2 group data, based on the readcount data obtained in the gene expression level analysis, DEGseq was used for differential transcript analysis, and Fold Change≥1.5 and FDR<0.05 were used as the screening criteria.
