# Supplementary material for: Influence of Polysaccharides From Polygonatum kingianum on Short-Chain Fatty Acid Production and Quorum Sensing in Lactobacillus faecis
Source: Front Microbiol. 2021 Nov 17;12:758870. doi: 10.3389/fmicb.2021.758870 (PMC8635744; doi:10.3389/fmicb.2021.758870)
Supplement: Supplementary file 1 [file Data_Sheet_1.zip › Supplementary Material 3.doc]

Supplementary Material 3

**(Proteome analysis method)**

1 Bacterial Protein extraction

Bacteria samples were collected for protein extraction as previously described. All samples were sonicated three times on ice in lysis buffer (8 M urea, 1% Protease Inhibitor Cocktail). After centrifugation, supernatants were collected and stored at 80 C until use. Protein concentrations were determined using BCA kit.

2 TMT labeling and HPLC fractionation

After digestion, peptide was desalted by a Strata X C18 SPE column (Phenomenex) and vacuum-dried. Dissolve the peptide with 1 M TEAB and label the peptide according to the instructions of the 6-plex TMT kit (Sigma).

The peptides were fractionated by high pH reverse-phase HPLC using Agilent 300Extend C18 column (5 um particles, 4.6 mm ID, 250 mm length). The operation is as follows: peptides were first separated with a gradient of 8% to 32% acetonitrile (pH 9.0) over 60 min into 60 fractions, and then the peptides were combined into 18 fractions and dried by vacuum centrifuging.

3 LC-MS/MS Analysis

Samples were reconstituted in 0.1% formic acid and total protein was analyzed by LC-MS/MS using EASY-nLC 1000 UPLC system. Autosampler directly loaded sample onto a home-made reversed-phase analytical column (15-cm length, 75 μm i.d.). A gradient processed was used for peptide separation: Solvent A was 0.1% formic acid and 2% acetonitrile, and solvent B was 0.1% formic acid in 98% acetonitrile, 0 ~ 26 min, 6% -23% B; 26 ~ 34 min, 23% -35% B; 34 ~ 37 min, 35% -80% B; 37 ~ 40 min, 80% B, all at a constant flow rate of 400 nL/min.

Mass spectra were acquired on Q ExactiveTM Plus (Thermo) equipped with a NSI source. The electrospray voltage applied was 2.0 kV. The m/z scan range was 350 to 1800 for full scan, and intact peptides were detected in the Orbitrap at a resolution of 70,000. The secondary MS scan range is set to a fixed starting point of 100 m / z and the secondary scan resolution is set to 17,500. The data acquisition mode uses a data-dependent scanning (DDA) program. To improve the effective utilization of the mass spectrum, Automatic gain control (AGC) was set at 5E4. Fixed first mass was set as 100 m/z.

4 Database search and bioinformatics analysis

The resulting MS/MS data were processed using Maxquant search engine (v.1.5.2.8). For the protein quantification method, TMT 6-plex was selected in Mascot. The false discovery rate (FDR) was adjusted to < 1% at protein, peptide and PSM levels.

Protein identifications were accepted if they could pass quality control. Gene Ontology (GO) analysis was performed using InterProScan (http://www. ebi.ac.uk/interpro/). Identified proteins domain functional description were annotated by InterProScan based on protein sequence alignment method, and the InterPro domain database was used. KEGG (Kyoto Encyclopedia of Genes and Genomes) analysis was used to annotate protein pathway.

The main point to note is that after the correlation analysis of the proteome data, it was found that the correlation between PS-2 and other samples was very poor (r=0.00~0.76), and the biological repeatability was poor (PS-1 vs PS- 2: r=0.11; PS-2 vs PS-3: r=0.34). At the same time, since the PS-2 sample was excluded from the transcriptomics data, in order to better parallel correlation analysis with the transcriptomics data and the rigor of the data, we excluded this sample in the subsequent differential analysis.
