# Supplementary material for: Influence of Polysaccharides From Polygonatum kingianum on Short-Chain Fatty Acid Production and Quorum Sensing in Lactobacillus faecis
Source: Front Microbiol. 2021 Nov 17;12:758870. doi: 10.3389/fmicb.2021.758870 (PMC8635744; doi:10.3389/fmicb.2021.758870)
Supplement: Supplementary file 1 [file Data_Sheet_1.zip › Supplementary Material l.doc]

Supplementary Material l

**(**[**Genomics**](C:/Program%20Files%20(x86)/Youdao/Dict/8.8.0.0/resultui/html/index.html#/javascript:;) **analysis method)**

1 Bacterial DNA extraction

After the strain was recovered, it was inoculated in liquid medium. The bacterial solution growing to OD_600_ of 0.5-0.6 was transferred to the centrifuge tube, and centrifuged at 5000 r/min for 5 min at 4℃, the culture solution was discarded; 5 mL of sterile 1 × PBS was added to the centrifuge tube to suspend bacterial, the centrifuge tube was centrifuged at 5000 r/min for 5 min at room temperature to remove the supernatant; repeat the washing (usually 2-3 times) until the supernatant is clear. Then, the bacteria were transferred to a large centrifugal tube, and place them in liquid nitrogen for quick freezing, and transferred to -80℃ refrigerator for storage.

Total DNA was isolated using the bacterial genomic DNA extraction kit (Cat. No. 13343, Qiagen, USA). The concentration and purity of DNA were determined by a Qubit fluorometer and a Nanodrop 2000 spectrophotometer (Thermo Fisher Scientific, Carlsbad, CA, USA). DNA integrity was assessed by 0.5% agarose gel electrophoresis.

2 Genome sequencing and assembly

The target strain genome was sequenced using a BGI platform at the BGI Health Co., Ltd. (Shenzhen, China).

Due to the large number of low-quality sequences, and error sequences in the original sequencing data, in order to obtain more accurate assembly results, a series of data processing systems is used to filter these impurity raw reads to obtain Clean Reads. A variety of software is used to assemble the sequencing data, Draft genomic unitigs, which are uncontested groups of fragments, were assembled using the Celera Assembler against a highquality corrected circular consensus sequence subreads set. To improve the accuracy of the genome sequences, GATK (https://www.broadinstitute.org/gatk/) and SOAP tool packages (SOAP2, SOAPsnp, SOAPindel) were used to make single-base corrections. To trace the presence of any plasmid, the filtered Illumina reads were mapped using SOAP to the bacterial plasmid database (http://www.ebi.ac.uk/genomes/plasmid.html).

Genetic prediction of assembly results using Glimmer software, tRNA, rRNA and sRNAs recognition made use of tRNAscan-SE (Lowe and Eddy, 1997), RNAmmer, and the Rfam database. The tandem repeats annotation was obtained using the Tandem Repeat Finder (http://tandem.bu.edu/trf/trf.html), and the minisatellite DNA and microsatellite DNA selected based on the number and length of repeat units . Use CRISPRFinder software to identify CRISPRs, get DRs and Spacers.

3 Gene annotation and protein classification

The protein sequences of predicted genes were compared with KEGG (Kyoto Encyclopedia of Genes and Genomes), COG (Clusters of Orthologous Groups), NR (Non Redundant Protein Database databases), Swiss-Prot, and GO (Gene Ontology), TrEMBL, EggNOG databases to obtain the annotated information.
